# AI-Enabled Sensor Fusion of Time-of-Flight Imaging and mmWave for Concealed Metal Detection

**DOI:** 10.3390/s24185865

**Published:** 2024-09-10

**Authors:** Chaitanya Kaul, Kevin J. Mitchell, Khaled Kassem, Athanasios Tragakis, Valentin Kapitany, Ilya Starshynov, Federica Villa, Roderick Murray-Smith, Daniele Faccio

**Affiliations:** 1 School of Computing Science, University of Glasgow, Glasgow G12 8QQ, UK; chaitanya.kaul@glasgow.ac.uk (C.K.); roderick.murray-smith@glasgow.ac.uk (R.M.-S.); 2 School of Physics and Astronomy, University of Glasgow, Glasgow G12 8QQ, UK; k.kassem.1@research.gla.ac.uk (K.K.); a.tragakis.1@research.gla.ac.uk (A.T.); ilya.starshynov@glasgow.ac.uk (I.S.); daniele.faccio@glasgow.ac.uk (D.F.); 3 Dipartimento di Elettronica, Informazione e Bioingegneria, Politecnico di Milano, Via G. Ponzio 34/5, 20133 Milano, Italy; federica.villa@polimi.it

**Keywords:** mmWave radar sensing, multi-modal sensing, information fusion, sensor fusion, mmWave, deep learning, metal detection

## Abstract

In the field of detection and ranging, multiple complementary sensing modalities may be used to enrich information obtained from a dynamic scene. One application of this sensor fusion is in public security and surveillance, where efficacy and privacy protection measures must be continually evaluated. We present a novel deployment of sensor fusion for the discrete detection of concealed metal objects on persons whilst preserving their privacy. This is achieved by coupling off-the-shelf mmWave radar and depth camera technology with a novel neural network architecture that processes radar signals using convolutional Long Short-Term Memory (LSTM) blocks and depth signals using convolutional operations. The combined latent features are then magnified using deep feature magnification to reveal cross-modality dependencies in the data. We further propose a decoder, based on the feature extraction and embedding block, to learn an efficient upsampling of the latent space to locate the concealed object in the spatial domain through radar feature guidance. We demonstrate the ability to detect the presence and infer the 3D location of concealed metal objects. We achieve accuracies of up to 95% using a technique that is robust to multiple persons. This work provides a demonstration of the potential for cost-effective and portable sensor fusion with strong opportunities for further development.

## 1. Introduction

Perception of a dynamic environment through a single (uni-modal) sensor inherently suffers from various limitations and vulnerabilities. For instance, consider a simple task of detecting the presence of objects in a room. A single RGB camera would be sufficient in many cases; however, such a system would be ineffective in low-lighting conditions or in scenarios where the target objects are occluded. For the task of identifying the presence of a concealed metallic object on an individual, an mmWave radar transceiver is a well-established choice. Objects illuminated by such Radio Frequencies (RFs) exhibit varying reflectivities based on their material composition [1,2,3]. In this respect, mmWave radars can sense objects in a scene that are occluded either due to the occlusion being transparent to these RF signals or the multipath nature of RF signals reflecting from diffuse surfaces [4,5,6,7,8]. Commercial RF transceivers, however, lack the ability to perform conventional imaging of a scene, which may be an important criterion in real-world use cases. A combination of multiple sensors with specific imaging characteristics provides complementary information about a scene; this makes multi-modal data acquisition setups highly desirable for many applications.

We propose a taxonomy of existing multi-modal information processing systems and argue that, within the context presented, they can be broadly categorized into two groups: structured and hybrid sensing. In the former, data acquisition systems generally comprise various camera setups, such as RGB, depth and Light Detection and Ranging (LiDAR) camera setups, which all spatially resolve the scene with a predetermined resolution. Such setups provide information about the structural nature of the scene in 2D and 3D. Hybrid sensing acquires complementary sets of data where the sensors can be combinations of cameras, LiDAR, Wi-Fi, radar, etc. For example, a depth camera and radar setup can acquire different properties about the scene; the data from one sensor complement the information provided by the other as they both capture different characteristics of what is being sensed without obvious overlap.

The technological innovation and commercialization of the security sector open the door to potentially invasive mass monitoring on a global scale. In particular, screening the general public for illicit weapons and devices introduces several complications which are exacerbated by human error and risk. Most modern metal detection schemes rely on electromagnetic induction, mmWave reflection or X-ray imaging. Metal detection scanning with an electromagnetic wand and performing pat-downs require time and risk the introduction of discrimination, and close-proximity monitoring is an inherent safety risk. The presence of walk-through metal detection scanners has become commonplace in airports, transport hubs, stadia and other public thoroughfares, and their use is growing among schools and other private sites. Whilst their performance ranks very high, they are not infallible. Together, their great cost and the tendency for such scanners to bottleneck people traffic in major thoroughfares, the result is a security solution which does not satisfy all use cases appropriately. The mmWave technology employed in airport security scanners has also long been under scrutiny for its ability to image through clothing, which has prompted privacy concerns [9,10,11,12]. There are methods of concealed metal detection which use thermal imaging cameras (based on the cooler thermal signature of metal objects concealed on subjects). Athena Security, Inc., have demonstrated a prototype dual-view-based concealed weapon detection approach using thermal cameras and RGB [13]; however, their current commercial systems do not appear to rely on this technology. Thermal sensors are typically two orders of magnitude more expensive than RGB/depth cameras of the same resolution. Furthermore, they require fusion with, e.g., an RGB camera, to discern more details of the scene, which breaks GDPR compliance. This suggests that there is an ongoing need for a technology that can remotely screen for metallic objects whilst still allowing people to maintain their freedom and privacy in public and without security bottlenecks and conventional image-capturing cameras.

In between these frequencies of operation sit mmWave imaging systems. Cheaper than X-rays, non-damaging to biological tissues and offering quasi-optical spatial resolution, mmWave imaging is also preferential for imaging humans over prolonged doses. At the core of this technology lies the principle of leveraging the distinct reflectivities of different materials when they are subjected to mmWave radiation. This modality allows for differentiation between materials, such as skin and metal, based on their varying reflection properties [14]. Additionally, mmWave signals can easily penetrate clothing [15].

The state-of-the-art mmWave-based solutions for metal detection rely on scanning gates. Several approaches have been developed that integrate mmWave scanning with AI (Artificial Intelligence) for improved detection fidelity. Evolv Technology has integrated AI with bespoke mmWave/optical active imaging to produce scanning gates that can process up to 900 people per hour, exceeding CT-scan (Computed Tomography) technologies [16]. IPVM claims that their AI can enhance existing gates and report a similar detection accuracy to Evolv’s bespoke system [17]. Sequestim Ltd. uses AI to enable passive walk-through scanning, i.e., seeing metal objects by the shadow they cast on the natural terahertz/infrared emission of the human body [18]. Despite the promise of AI in concealed metal object detection, there is still room for improvement. Reported incidents of undetected knives brought into schools through AI-enabled scanning gates have raised questions over their efficacy [19,20,21,22,23].

Recent works have shown that it is possible to accurately detect the presence of concealed metal on a person in a scene using only an mmWave radar transceiver [24,25,26,27]. A common limitation is the requirement to scan a person, which may not capture the intricate details of the structure and spatial properties of the scene.

In this work, we avoid the pitfalls of uni-modal radar sensing by adding structural guidance to the system through a depth sensor. We propose and demonstrate that adding this additional structural guidance in the feature space is essential for adding spatial context to the task of AI-assisted metal object detection. We collect multiple multi-modal datasets with radar and multiple depth cameras to show that our technique generalizes across various permutations of radar and camera, and is not dependent on any particular combination of sensors. Finally, we propose a late-feature fusion model that first extracts features and then embeds them into a high-dimensional latent space to create representations of the multi-modal data. Following this, the model learns to optimize a combination of them together using back-propagation to detect the presence of a metal object. Our experiments demonstrate the ability of multi-modal sensor fusion to be used as an effective detection system compared to single-radar setups.

## 2. Methods

**Figure 1:**
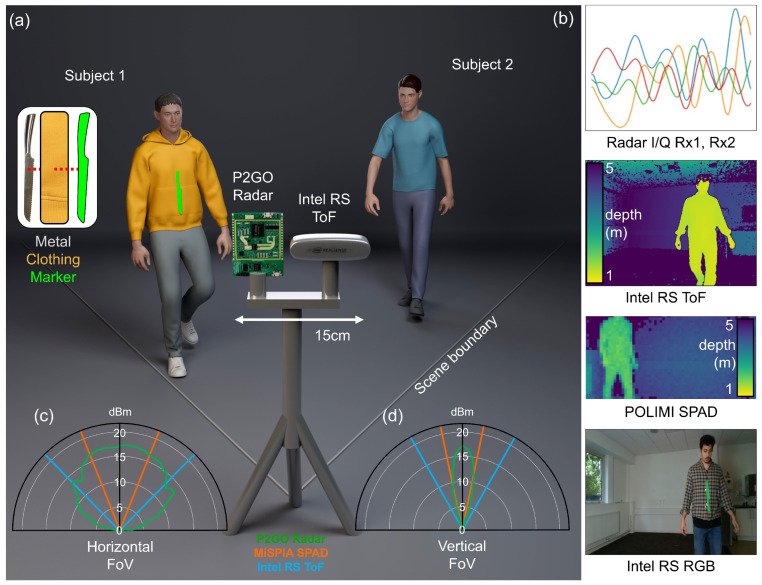
Experimental setup for radar and depth-camera-based concealed metal object detection. The setup in (**a**) shows one or two subjects walking in view of the devices with one subject concealing a knife beneath their first layer of clothing. The knife location is labeled with a green paper marker for training using the RGB camera data. The acquisition data modalities are listed in (**b**), specifically, the intermediate-frequency radar signal (where colour indicates the real and imaginary parts of the two channels), depth (either Intel RealSense or POLIMI SPAD camera) and RGB color images. The relative field of views (FoVs) for each device are shown in (**c**,**d**) on both horizontal and vertical axes. Intel RS RGB image and all depth maps throughout depict the authors, with permission. Three-dimensional render courtesy of Diana Kruhlyk.

Consider the setup shown in Figure 1: The radar and 3D depth camera are paired together before a static scene (approximately 5×5 meters). The former is an Infineon Technologies (Neubiberg, Germany) Xensiv Demo Position2GO 24 GHz radar transceiver with 1 transmitting and 2 receiving antennas capable of tracking multiple targets at up to ∼20 m. It uses fast-chirp FMCW radar technology, the specifications of which are listed in Table 1 for reproducibility [28,29,30]. The latter is an Intel Corporation (Santa Clara, CA, USA) RealSense D435 stereo depth camera which uses a pair of ultrawide sensors 50mm apart to calculate depth from stereo images. Furthermore, the Intel D435 includes an integrated colour (RGB) sensor which is co-registered to the depth data as a reference for training only [31]. This was chosen to provide a complementary sensing modality to the mmWave transceiver whilst still providing a form of GDPR compliance due to depth images containing far fewer identifying features than conventional RGB CMOS camera images.

To investigate the potential of SPAD (Single-Photon Avalanche Diode) array technology, we also replaced the Intel depth camera with an indirect Time-of-Flight (iTOF) SPAD camera, developed at the Politecnico di Milano (POLIMI) [32,33,34]. This SPAD camera generates a 64×32 resolution depth map of a scene with a $20^{\circ }\times 40^{\circ }$ field of view, which more closely matches that of the radar used. This SPAD-based solution opens the door for ultra-low-light depth sensing, and different modes of operation could also provide access to the time dimension for single-photon applications.

### 2.1. Experimental Procedure

During data acquisition, subjects removed all metals from their person and concealed a 20 cm long steel knife underneath their first layer of clothing, specifically, on the chest. The location of the knife was labeled by affixing a green paper marker on top of clothing. This could then be detected only in the RGB reference image and was not spatially resolved in the depth image. The procedure used for data collection and deployment of the metal detecting system was born out of our previous work on generating 3D spatial images using temporal data [5,6]. Here, a radar transceiver and depth camera pairing was used to gather data for a human subject moving through a static scene.

The experiments were conducted with a sensor rig that was activated to stream simultaneously whilst the subjects moved through the space at walking pace for between 2 and 6 sets of 3000 frames (limited by RAM storage limitations) at ∼20 Hz. These datasets included various configurations, e.g., subject with/without object concealed or multiple subjects. For simplicity, the subject always faced the sensor rig, and their randomized movements were intended to encompass all likely positions and velocities. All three data streams were then saved to file before post-processing and training. During deployment of the trained system on test data, only the radar and ToF depth maps were used. We then input the depth image and raw radar I/Q signals to our dual-input neural network to predict segmentation masks.

### 2.2. Network Architecture

**Figure 2:**
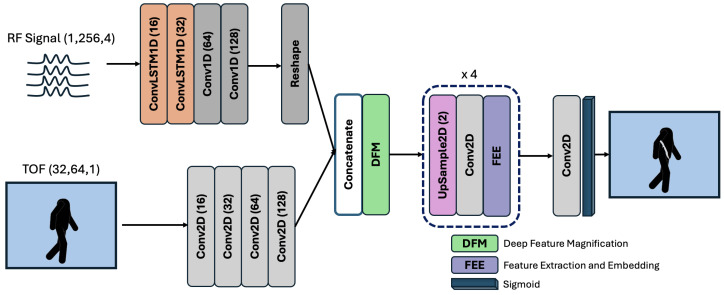
Our neural network architecture, $mmSense_{AF}$, for concealed metal detection. We process the radar data as sequences using convolutional LSTM blocks and create embeddings from the spatial depth image using convolutional blocks with large receptive fields. After concatenating the embeddings from both modalities, we extract joint concepts from them using the deep feature magnification block. We then use a convolutional decoder coupled with a feature extraction and embedding module to upsample this encoding to generate the output mask.

Our neural network is presented in Figure 2. Firstly, we created a dataset $\left\{X_{i},X_{r},Y\right\}$, where $X_{i}$ are the input Time-of-Flight (ToF) images, $X_{r}$ is the input radar data and *Y* is the corresponding binary segmentation mask denoting the location of the knife in the ToF image. All SPAD ToF images were used in their native 32×64 resolution. The RealSense ToF images were downsampled from their native resolution to 48×64. Following this, we replaced 0-depth pixels with the minimum pixel value in the scene to create a smooth image without discontinuities, which can cause gradient instabilities in training the neural network. After this, we standardized the images by first subtracting the mean pixel value and then normalizing the image with the maximum value. The radar data were collected and input to the network in their native I/Q basis, normalized in the range of [0, 1]. Edge computing scenarios demand the most efficient data processing methods, which suggests some pre-processing of the radar data is beneficial. In our previous related work [6,24], we used computed Range profiles for 3D scene reconstructions (mmWave and ToF) and Amplitude-Range Doppler (ARD) plots for detecting concealed metals. Our experience in this work, however, was that these processed domains do not offer a statistically significant time or result in an advantage over raw I/Q data when training the network. While Range and Range-Angle are sparser representations, they are ordered vectors; in contrast, I/Q is a continuous, largely translation-invariant basis. This makes the latter better suited for the convolutional layers used in our model. Comparisons can be made to positional encoding in large language models; instead of feeding raw token positions to the network, we encode them into Fourier harmonics, creating a continuously varying high-dimensional space. This encoding approach parallels how we see the I/Q data functioning within the network—it provides a richer, more flexible input for learning.

#### 2.2.1. Implementation

Our model was trained using TensorFlow (Google LLC, Mountain View, CA, USA) 2.12.0 for a maximum of 100 epochs. We used a batch size of 64 for all our experiments. We used the Adam optimizer with an initial learning rate of 1E-3, which we then reduced on a plateau to a minimum value of 1E-6. We ran all our experiments on one NVIDIA Corporation (Santa Clara, CA, USA) A5000 GPU. All weights for our model were initialized from a random normal distribution. We used binary cross-entropy loss to create a measure for the difference between the distributions of the predicted and ground truth labels.

#### 2.2.2. mmSense_*AF*_

**Figure 3:**
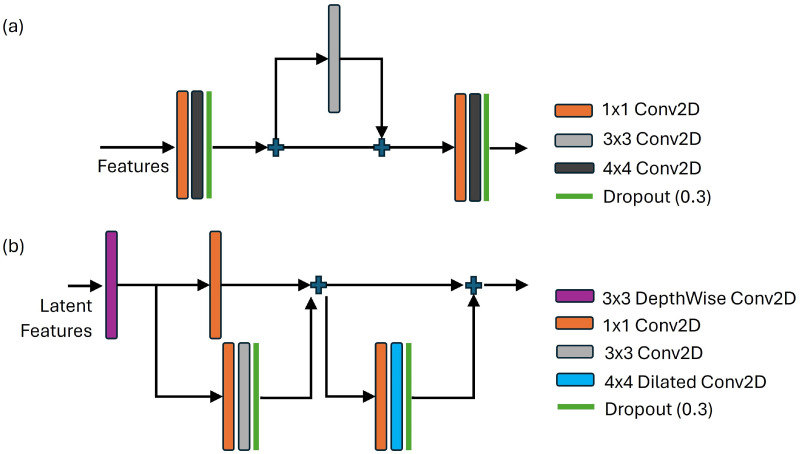
(**a**) The structure of the deep feature magnification block. This takes the concatenated features from both modalities as an input and learns the relation between them by focusing on relevant features using increasing convolutional kernels and receptive field sizes in the convolutional block. (**b**) The feature extraction and embedding block. This processes the upsampled latent features with increasing convolution kernel sizes in order to learn to correlate the location of the concealed object with the depth features, achieved by processing the encodings at varying receptive fields of the convolution kernel.

Our neural network architecture, called $mmSense_{AF}$ (Auto-Focus), is a dual-channel convolutional encoder–decoder structure that iteratively learns to focus on the combined latent features from the radar and ToF modalities. This is in order to extract the 3D location of the radar mask in the ToF image. It consists of distinct encoders for both modalities, and processes them based on the properties of the individual mode; it extracts features from the radar signal and ToF images and outputs their latent representations. Following feature extraction, the two embeddings are concatenated across the depth axis to create a latent representation, which serves as a joint embedding of the data. We process this joint embedding to create a true latent space of combined radar and ToF features using the deep feature magnification block (DFM), as shown in Figure 3a. The outputs from the DFM are then upsampled and passed through a convolution layer to generate upsampled features. We then process these features using a feature extraction and embedding (FEE) block (Figure 3b). The output from the last FEE block is passed into a convolutional layer with a sigmoid activation function to generate a per-pixel probability of the location of the concealed metal object in the ToF scene.

Radar Encoder. The radar encoder consists of two ConvolutionalLSTM1D layers followed by three Conv1D layers. We pass a 1×256×4 radar input to the the first ConvolutionalLSTM1D layer to extract time-dependent features from the input data sequence. The second layer further processes these data, and converts the sequences into convolution-compatible features. Using ConvolutionalLSTM layers on such data sequences has been shown to alleviate the redundancy of fully connected layers inside LSTM layers due to the ability of convolutional kernels to share weights while providing a better spatio-temporal feature representation of the data. Previous works such as [35,36] demonstrated this quality extensively for multiple wearable sensors. The following two convolution layers then create high-dimensional embeddings of the data using a stride of 2 to simultaneously reduce the feature map size. All convolutional kernels in this layer have a large receptive field of (1, 7) in order to incorporate more time-dependent information, and a larger spatial context to compute the features for the next layers. The third convolution layer is used to learn the final embedding of the radar data. The output filter maps 16→32→64→64→128. All layers use a ReLU activation function. The output from the last Conv1D layer is reshaped to resemble the output shape of the ToF features.

ToF Encoder. The ToF encoder consists of 4 Conv2D layers with zero padding and the ReLU activation function. Each convolutional layer uses strides of varying receptive fields to create a reshaped latent representation of 4×4×128 features, making it compatible with the reshaped radar features. We use large receptive fields of size (7, 7) in this encoder as depth images tend to be smoother than RGB images and have fewer intensity transitions. Further, given that our goal is to learn global properties of the scene, and not local features about the individual in the scene (which are not visible in a ToF image), a large receptive field allows us to capture the relation of the large objects in the FoV of the camera. The output filter maps 32→64→64→128.

Latent Fusion. We coarsely combined the radar and ToF latent features via a simple concatenation operation. We then passed this joint embedding into the DFM block [37]. The DFM block magnifies the relationships between the features of the two modalities. The concatenated features are first passed through a DepthwiseConv2D layer. This first learns individual features for all 256 concatenated filter maps in the feature representation with a spatial receptive field of 3×3, followed by a 1×1 convolution across the depth of the features, to aggregate information from both modalities into a learned joint representation. These features are then incrementally processed by convolutions of varying receptive fields, 1×1, 3×3, 1×1 and 4×4 to create spatially consistent representations, along with extracting any subtle feature dependencies that exist between the representations of both modalities. We also used a 4×4 convolution with a dilated receptive field to learn larger spatial relationships between the data, which facilitates the interlinking of dynamic structures in the feature space across a larger region, providing larger global context to the features. We used dropout layers with a rate of 0.3 throughout to prevent overfitting. All convolution layers in the DFM block use BatchNormalization followed by the ReLU activation function.

Decoder. We decoded the latent fusion output by incrementally upsampling it by a 2×2 factor and processing the upsampled output with a convolution layer. These features are passed to the FEE block [37] to extract further spatial correlations in the features at the different upsampled scales. The FEE block performs consecutive 1×1 and 4×4 convolutions on the features. Similar to the DFM block, having a small 1×1 receptive field builds local features across all filter maps while keeping the parameter size of the model low. Following this with a large 4×4 receptive field size aggregates the features along with a larger spatial context. As the location of the hidden object is only a few pixels in size, extracting small receptive field features across depth and aggregating them with a larger spatial context aid the localization by looking at the features in multiple receptive fields. Similar to in DFM, we used dropout layers with a rate of 0.3 throughout to prevent overfitting. All convolution layers in the FEE block use BatchNormalization followed by the ReLU activation function. The output from the last FEE block is processed by a convolution operation with a receptive filed of 1×1 and a sigmoid activation function to generate per-pixel probabilities for the presence of a concealed object on the person in the scene.

## 3. Results

**Figure 4:**
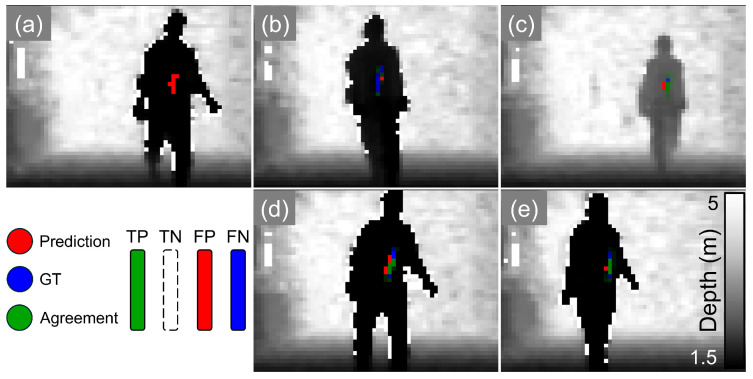
ToF 1P (Wide FoV) visualizations. Prediction (red) and ground truth (blue) are overlayed to depict agreement (green), which represents the standard F-scores in each frame. (**a**) High False-Positive rate, (**b**) high False-Negative rate, (**c**,**e**) high agreement, (**d**) translated prediction. The shown images are samples of video frames from a test set which comprises a prediction mask overlayed on the original depth frame for the P2GO radar and the Intel RealSense depth camera with one person in the scene. Visualization 1 in the Supplementary Materials.

**Figure 5:**
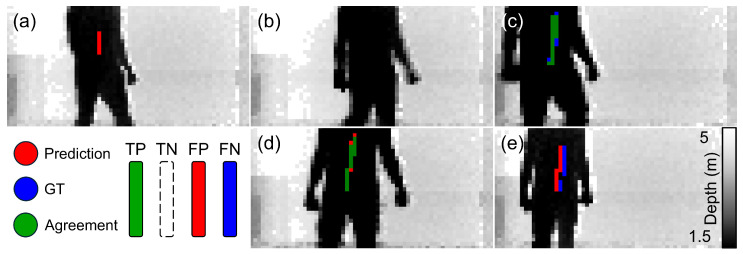
SPAD 1P visualizations. (**a**) High False-Positive rate, (**b**) negative label, (**c**,**d**) high agreement, (**e**) translated prediction. The shown images are samples of video frames from a test set which comprises a prediction mask overlayed on the original depth frame for the P2GO radar and the POLIMI SPAD camera with one person in the scene. Visualization 2 in the Supplementary Materials.

**Figure 6:**
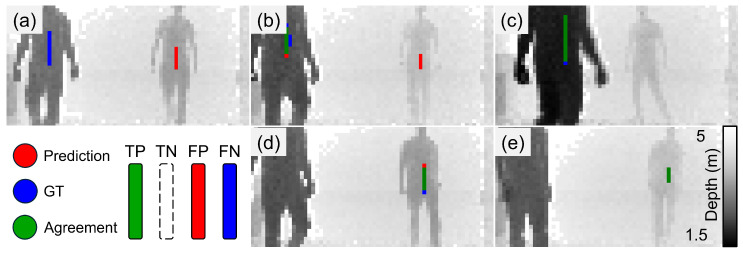
SPAD $2P_{2}$ visualizations. (**a**) Mislabelling, (**b**) semantic issue, (**c**–**e**) high agreement. The shown images are samples of video frames from a test set which comprises a prediction mask overlayed on the original depth frame for the P2GO radar and the POLIMI SPAD camera with two people in the scene. Visualization 3 in the Supplementary Materials.

We demonstrate the effectiveness of our system quantitatively in Table 2 and qualitatively in Figure 4, Figure 5 and Figure 6 (still frames from Supplementary Videos S1–S3, respectively, with the acquired datasets and architecture publicly available in [38]). We also show the necessity of sensor fusion to integrate spatial information into the feature processing through our results in Table 3. We report standard F-score metrics, namely, accuracy (agreement), sensitivity (True-Positive (TP) Rate), specificity (True-Negative (TN) Rate) and precision (Positive Prediction Value), of our model’s ability to predict the presence of a concealed metal object (knife) in a 3D scene. We define agreement with the ground truth (GT, shown in green) when the central weight position of the prediction is within five pixels of the GT position. Here, rows 1–3 present the values for the POLIMI SPAD camera and the P2GO radar: row 1 probes the concealment from one person; row 2 assumes a metal object is present and identifies which person has it; row 3 combines both concealment and identifying the person. Row 4 shows the same test as row 1 but for the Intel RS depth camera instead, which has a larger field of view and extends to regions where the P2GO radar weakens in strength. The reduction in performance when comparing the SPAD and ToF sensors can be attributed to the 2 dBm versus 4 dBm fall-off in radar antenna strength shown in Figure 1, which drops to 7 dBm in the edge data. Careful pairing of modalities to account for this should mitigate the effect in future widefield applications. We report strong detection capabilities for the single-person case and promising values in the multiple-persons case.

Table 3 demonstrates the value in fusing the two modalities together. We note that an accuracy of 50.4% using just the depth modality understandably amounts to a random guess as the concealed metal is not visible in the spatial domain and the trained network cannot distinguish between metal and non-metal training data. Care was taken to ensure that the metal was thin enough and flush against the body to not appear as a protrusion in the depth data—its relative prominence from the body was less than the depth resolution at the subject distance—in order to represent true concealment. The depth camera on its own has zero capability to distinguish between scenes with and without a knife; its role is to enhance the detecting capabilities of the mmWave radar. The radar+depth result neared 95% compared to 74.6% for the radar alone. We also show this comparison for SPAD2P2, which shows that the radar+depth result (70%) outperformed only depth (50.2%) and only radar (60.5%). These results demonstrate that the depth image contributes little towards the metal detection but serves as a discriminator for the network to correlate spatial information with the radar data to train on the radar data more effectively.

Figure 4, Figure 5 and Figure 6 visualize the results in the one-person and two-person cases when pairing the Infineon P2GO and the POLIMI SPAD camera as our data acquisition setup. We show visualizations of high agreement and high true- and false-positive results, as well as predictions where the presence of the metal is translated in the image but the structure of the metal is predicted correctly.

## 4. Discussion and Conclusions

In this work, we have demonstrated the first promising results towards 3D remote detection of concealed metal objects using radar and depth data fusion. We proposed a feature fusion and processing framework based on a deep encoder–decoder neural network architecture capable of extracting relevant features for multiple receptive fields of the input. This was used to locate the presence of concealed metals in a 3D scene. We have demonstrated the effectiveness of such a system for static scenes containing single and multiple subjects.

The novelty in this work lies not with sensor fusion in and of itself; instead, it is by combining mmWave and depth sensing modalities through sensor fusion that we can improve the capability of our previous work [24] to a standard that demonstrates its applicability in real-world scenarios. In such scenarios, a cheap and portable mmWave transceiver with only a few antennae cannot perform single-shot concealed metal detection at range when multiple subjects occupy the scene due to the ill-posed problem of RF signals from multiple locations sharing the same range bins.

In the mmWave regime, cost-effective transceivers such as that used in this work can be calculated as 1D range plots through a Fast Fourier Transform. With several transmitting and receiving antennae, it is also possible to determine angle and velocity against range. Alone, this amounts to being able to determine if there is an object in the radar’s field of view with a detectable radar cross-section. In our previous work [24], we demonstrated that machine learning can be performed on training data to determine if an object (a person) contains a higher-than-expected reflectivity in mmWave.

However, the resolution of the calculated complex range Doppler plot is effectively only a few spatial pixels—this is insufficient to handle more complicated scenes, such as those with multiple subjects, occluding environmental objects which produce their own unique reflectivity and subjects in different poses. To build upon the state of the art, we combined the established mmWave and depth modalities to better match the anticipated use cases: public thoroughfares, businesses and transport stations. Our work is chiefly about showing that the limited use case of a single cheap radar transceiver can be expanded greatly by sensor fusion with a modality which cannot detect concealed metal but can discern object geometries, depths and poses for any number of complicated scenes.

Some key modifications were made to the fusion processing model as this investigation progressed in order to enhance its performance. Note that we intentionally fed the raw, synced I/Q and depth data as inputs to our model. Employing a deep-learning-based feature fusion methodology helped to create joint representations of the two features. We created a ‘convolution-heavy’ encoder–decoder model for our task due to the ability of such layers to be trained end to end to extract features along with output-per-pixel probabilities for locating the concealed metal on the subject. This allowed us to train a model that can go directly from raw data to predictions without the need for any extensive pre-processing of the data as deep neural networks have been extensively applied in such training regimes and have demonstrated success.

We initially started with a fully convolutional encoder–decoder structure with a dual branch encoder (one branch for RF data processing exclusively by 1D convolution layers, and another for the depth data built with 2D convolution layers with large 7 × 7 kernels), creating an addition-based fused latent representation of 4 × 4 × 128 features. We then processed this with a series of 2D upsampling and 2D convolution layers to predict a single-channel output mask of probabilities equal to the size of the depth image, detecting the presence of concealed metal. These probabilities were then thresholded using a value of 0.5 to obtain a binary mask, which we compared with the binary mask generated using the chromakey labels for the concealed metal to train the initial network using back-propagation.

Based on the existing literature [36,37], we observed that ‘RF-like’ data with multiple channels benefit from being processed by layers such as convolutional LSTMs as they replace the matrix multiplication between feature maps and LSTM layers with convolutions. This allows for faster processing of the data, along with improved learning of spatio-temporal dependencies in the data (also cited in the manuscript).

The need for adding the deep feature magnification (DFM) block and the feature extraction and enhancement (FEE) block arises from the fact that, while upsampling the latent representation of the data, a larger global context always helps to create better global features of the data when going from data representation to an image such as a mask with a concealed metal location. We embedded this concept into our network using the DFM and FEE blocks, which employ varying receptive fields in the convolution kernels via different dilation rates—this can be seen as a way of incorporating a high-level structure into the features as we upsample to the mask. This allows us to incorporate high-level structural properties into the features, while the 1 × 1 convolutions in both blocks process per-point features to add local context along with this. Experiments undertaken, for instance, removing the DFM block (which processes the latent feature representation) demonstrated this. On removing the DFM block from our model (and keeping the other components constant), the overall accuracy of the model for the SPAD1P case dropped from 94.5% to 84.3%, while adding the DFM back to the model and removing the FEE layer reduced the overall accuracy to 85.7%. This shows how important it is to process the latent features incorporating global features into them to create a better decoder for our problem.

It is also important to underline the considerable challenges faced when generalizing this technique to a wider range of scenarios. Firstly, we chose to focus only on detection within a static, fixed scene. This greatly simplified the problem by limiting the variables to the subjects and the presence of metal objects on their person. In order to generalize to different background scenes, a much larger training dataset is needed. The number of participants in the scene was also constrained to one or two as the transceiver used features such as 1Tx and 2Rx antennas and an equivalent depth resolution of >70 cm—more subjects in the scene would convolute the signal received and make identification more difficult. Alternative transceivers with greater angle and depth resolution are available, which would allow for up to five subjects in the scene to be tracked through radar alone [39,40,41,42].

We also note the impact of the specular reflections of the radar signal from the metal objects. We found that flat-surfaced metals tended to reflect the signal without any discernible scattering. A consequence of this was the ability to detect flat metal objects at distances of multiple meters due to the higher signal-to-noise ratio, but only when the surface normal of the flat metal surface and the radar optical axis deviated by less than 5–$10^{\circ }$. To maintain simplicity and relevance for this proof of concept, we ensured that the subjects faced the detector throughout the acquisition (akin to use cases in a public thoroughfare), and selected a steel bread-knife as the metal object due to its multiple angled surfaces and its relevance to real-world concealed carry. These challenges may be mitigated by multiple separated detectors, or curved detection rings which provide multiple viewpoints to the subjects. Our previous study [24] investigated metal objects of varying size and curvature, and demonstrated that such a system does not detect keys and smartphones in the pockets. In future studies, it is pertinent to generalize the system to the full range of potential concealed objects in terms of shape, size, placement on the body and classification between threats and benign metals.

This work seeks to highlight the untapped potential of portable commercial radar and depth sensing technology for metal detection. The approach provides a discreet, cost-effective and safe means to deploy metal detection in public and private spaces. We have observed that, provided there is sufficient training data and availability of powerful AI models, the potential exists to generalize this concept to wearable technology which can run in real time with background independence, which could be part of a step change in the surveillance and security of the future.

## Figures and Tables

**Table 1:** Specification and settings used for the radar transceiver. The full range of values is quoted from the manufacturer user guide [30].

Specification	Value and Range
Radar model	Infineon Demo Position2GO
Bandwidth	24–24.25 GHz
ADC sampling	300 μs [50:3000 μs]
Slope	0.83 MHz/μs
Chirps/frame	1 [up to 16]
Samples/chirp	256 [32,64,128,256]
Down chirp, standby	100 μs
Frame rate	2000 μs
Min/max distance	1 m/20 m (12 m, human subjects)
Range accuracy (>0.6 m)	±0.2 m
Range resolution	0.9 m
Field of view	Hz 76°, Ve 19°
Angle accuracy	≤5° for ±30° and ≤ 10° for ±65°

**Table 2:** Our results table provides quantitative results on the different data acquisition regimes in terms of standard F-score metrics. All results are generated using our latent space feature fusion model with the Infineon P2GO radar and a depth camera (SPAD refers to the POLIMI camera and ToF Wide FoV refers to the Intel RealSense D435).

Regime	Accuracy (%)	Sensitivity (%)	Specificity (%)	Precision (%)
SPAD 1P	94.5	93.4	93.8	96.6
SPAD $2P_{1}$	85.6	91.0	61.6	92.2
SPAD $2P_{2}$	70.0	70.4	69.6	74.6
ToF 1P (Wide FoV)	67.4	64.4	75.6	60.3

**Table 3:** Demonstrating the need for sensor fusion. Our results demonstrate that fusing the spatial and radar modalities allows us to learn correlations between both domains, creating a more accurate regime for concealed metal detection.

SPAD 1P	Accuracy (%)
Depth Only	50.4
Radar Only	74.6
Radar+Depth	94.5
**Radar+Depth (TOF 1P)**	67.4
**SPAD 2P2**	
Depth Only	50.2
Radar Only	60.5
Radar+Depth	70.0

## Data Availability

The data underlying the results presented in this paper are available in [38].

## Supplementary Materials

The following supporting information can be downloaded at https://www.mdpi.com/article/10.3390/s24185865/s1, Video S1: ToF 1P (Wide FoV) visualizations. Video S2: SPAD 1P visualizations. Video S3: SPAD $2P_{2}$ visualizations.
